# The Dependence on Smokeless Tobacco in the South Asian Communities in East London

**DOI:** 10.5539/gjhs.v8n1p194

**Published:** 2015-05-15

**Authors:** Amjad Hussain Khaja, Abdulsalam Ali Zwiad, Bassel Tarakji, Giath Gazal, Feras Albaba, Nader KalajI, Waleed Petro

**Affiliations:** 1 Department of Oral Maxillofacial Sciences, Alfarabi Colleges, Riyadh, Saudi Arabia; 2 Faculty of Dentistry, Sanaa University, Sanaa, Yemen; 3 Oral and Maxillofacial Surgery, College of Dentistry, Taibah University, Saudi Arabia; 4 Depatment of orthodontics, Faculty of Dentistry, Aleppo, Syria; 5 Department of Restorative Dentistry and Prosthodontics. University of Science and Technology, Sanaa, Yemen

**Keywords:** smokeless tobacco, South Asian, tobacco dependency

## Abstract

**Background & Objective:**

The purpose of the study was to understand the dependency on smokeless tobacco.

**Methods:**

The major aspect of the interview was to study the type of chewing tobacco used, frequency of purchase of chewing tobacco, change in attitude and behavior after the use of chewing tobacco. This study was done in 2005 in London. Of the 110 respondents interviewed 88 were used for the data analysis.

**Study Design:**

An exploratory study was conducted in East London, United Kingdom. The selected sample was interviewed through a questionnaire, based on the Severson Smokeless Tobacco Dependence Scale.

**Results:**

Cross tabulations report that in a sample of 88 South Asian UK resident men 46.6% used leaf (paan), 43.2% used processed form of chewing tobacco and 10.2% used gutka. Older age (67%) respondents were more likely than the younger age (30%) respondents to chew tobacco. The frequency of purchase of chewing tobacco is reported high (67.2%) in the older age group than the younger age group (50%).

**Conclusion:**

This current study used an amended form of the Severson Smokeless Tobacco Scale questionnaire to study the dependency on smokeless tobacco. The study could be developed in the selection of the sample, which would include both males and females to study the dependency on smokeless tobacco.

## 1. Introduction

Different forms of smokeless tobacco are used in the minority ethnic groups, particularly among the South Asian population. Of the three South Asian groups, Bangladeshis (both men and women) were by far the most likely to report chewing tobacco: 19% of men and 26% of women, compared with between 2% and 6% for Indian and Pakistani men and women. Tobacco is often consumed in combination with other products. Betel leaf is used to wrap the fillings to form a quid. The leaf has a mint flavor and is considered a mouth freshener. The leaf (paan) itself is relatively harmless the health risks arise from the tobacco and other ingredients contained in the paan. Ready-made mixtures of snuff are known as Gutka or paan masala which are chewed either on their own or in betel quid. They are prepared by baking and curing a mixture comprising areca nut, lime, spices and tobacco (The Health of Minority Ethnic Groups, 1999).

### 1.1 Smokeless Tobacco Described

Smokeless tobacco is a type of tobacco or a tobacco blend which instead of smoking is chewed or inhaled. Smokeless tobacco is available in three forms.

Chewing tobacco comes in the form of loose leaves or twists and as the name suggests it is chewed. Snuff is available in the form of dry or moist leaf finely ground or shredded and is available in pouches. This form of tobacco is used by placing a pinch of snuff between the cheek and the gum or is inhaled through the nostrils.

Betel quid is usually found in Asia (India), Africa it is commonly known as the pan or pan masala. It is a traditional method of using tobacco. It consists of lime (calcium carbonate), tobacco, areca nut, and flavoring agents and catechu. Betel quid is used by placing between the gum and the cheek and is slowly chewed and sucked (Smokeless Tobacco: Addictive and harmful, 2004).

### 1.2 Previous Studies

Previously there were a number of reviews and studies conducted on various aspects of smokeless tobacco. These reviews describe the various aspects of the smokeless tobacco the pattern of use, measuring the dependence of smokeless tobacco users, smokeless tobacco as addictive drug, use of smokeless tobacco among the youth and addiction, prevention and treatment of spit tobacco. But majority of these studies refer to the smokeless tobacco the studies are conducted in US and North America and aims the people who live there where smokeless tobacco is used in the form of moist snuff or spit tobacco. But the literature content is less when discussing about the various aspects of traditional smokeless tobacco (paan) in South-Asian communities. One of the reviews conducted in East London to study the prevalence of paan chewing with tobacco by UK-resident Bangladeshi women and the extent to which they manifest nicotine dependence.

The cross sectional study was conducted at two local authority housing estates in Tower Hamlets, East London. Participants were 242 Bangladeshi women, Selected at random from the current electoral register, who supplied a saliva sample for cotinine and an expired air sample for carbon monoxide analysis (Croucher et al., 2003). They also participated in a structured interview assessing knowledge, attitudes and behaviour with respect to tobacco use. Smoking prevalence was low, but the prevalence of paan quid with tobacco chewing is high in this sample of Bangladeshi women. Cotinine concentration appears to be a reliable indicator of levels of nicotine dependence among paan quid with tobacco chewers. Questionnaire-derived items can be used to identify those with above-average levels of nicotine dependence (Croucher et al., 2003).

### 1.3 Using the Mosques to Advice

Mosques have been a significant place either for collection of sample for a study or to deliver health promotion and health awareness messages. This study was done in 2011 in London. The studies discussed below are few such studies conducted previously, which have used mosques for such purposes. Islamic Medical Association of Uganda, Kampala in 1992 have designed an AIDS awareness prevention project known as Family Acquired immunodeficiency Syndrome (AIDS). Education and prevention through Imams (Kagimu et al., 1995). Community-based malaria control project covering predominantly Muslim population in the United Republic of Tanzania. Education on this subject was provided in the mosques during the Friday noon religious services when the attendance levels are relatively high (Mfaume et al., 1997). The purpose of this study was to elucidate the roles of imams, Islamic clergy, in meeting the counseling needs of their communities as the Muslims have experienced increased stress since September 11, 2001.

Imams reported that their congregants came for religious or spiritual advice and reported increased need to counsel persons for discrimination. Imams were told to meet the need of the congregants though they had little formal training in counseling.

## 2. Methods

The key features of the study planning included assembling the questionnaire and piloting the questionnaire. An ethical clearance was obtained from the institutional review board 1654-2004/05 Queen Mary, University of London. The basic principle of planning a study is to lay out the objectives of the investigation. The objectives should be clear and unambiguous. The concepts of the questionnaire are to be clearly defined and phrased unambiguously. During planning a questionnaire attention should be given towards the length and phrases used in the question. Long questions and difficult words can lead to inattention, refusals and incomplete answers. The questionnaire had two sections. The first section collected general introduction about the individual interviewed questions were asked on like age, place of birth, whether he chewed or smoked tobacco on a regular basis and type of chewing tobacco used. The second section specifically deals with dependency on the smokeless tobacco. The original Severson Smokeless Tobacco Dependence Scale questionnaire has a question do identify how many days does a tin or pouch of chewing tobacco lasts this question has been amended as how many days does the purchase lasts to reflect the South Asian tobacco use. The questionnaire was originally written in English.

As the study sample was the south Asian communities it was decided to translate the questionnaire into the local languages (Urdu and Bengali) to compare the data of the items in questionnaire and to have an equality and uniformity in each language. The questionnaire was translated into Bengali and Urdu with the help of English speaking bi-lingual translators with in the local community. The translated questionnaire were again second translated into English and the two versions were compared, sometimes there is a possibility of inaccuracy of the translated questionnaires due to errors in exact translation of English terminology hence a second translation is necessary to avoid inaccuracies (Bhopal et al., 2004). The commonly used non-random method is quota sampling, in which a pre-defined number of people who meet certain criteria are selected (Steward, 2002). In this sample one hundred and ten adult males were selected from different population of the south Asian communities. The study design is vitally important as poorly designed studies may give misleading results. This study is an exploratory study.

The questionnaire was piloted to the individuals to be interviewed. Adult males coming to the local mosques to offer prayers were recruited into the study. The imams, head of the two local mosques were approached to question permission prior to the conduct the study. The imams were shown the questionnaires in all the three languages and a brief introduction was given to them regarding the purpose of the study, as their help and permission was important. After getting the approval from the imams the individuals were interviewed individually. A focus of attention was given to Fridays as important prayers are offered on Friday and the attendance at the mosques is significantly more than on other days. Individuals were approached to help interview the individuals in the local language especially Bengali. The individuals were given a brief introduction about the study conducted and the individuals were interviewed. After the completion of the interviews the next phase was the data entry and data analysis.

The collected data was coded on a Master Coding Sheet using MS-EXCEL. The data was analyzed using the SPSS Statistics a software package used for statistical analysis.

## 3. Results

Data was collected on twelve days in the months of July and August 2005. Mosque attendees were selected opportunistically and invited to take part. One hundred and ten agreed to do so. Of these twenty two were not included in the analysis because they were either smokers only (8) or non- regular users i.e., more than twice a week, chewers of tobacco products (14). Therefore reports 88respondents who met the inclusion criteria for the entry into the study. Of the eighty eight 39 were interviewed in Urdu, 29 were interviewed in Bengali and 20 were interviewed in English. The mean age was 45.3 years with a range of 25-67 years. Twenty-five were born in Pakistan, 29 in India and 34 in Bangladesh. Cross tabulations report that in a sample of 88 South Asian UK resident men leaf (paan) was most used form of chewing tobacco (46.6%) followed by processed form of chewing tobacco (43.2%) and gutka (10.2%). Older age (67%) respondents were more likely than the younger age (30%) respondents to chew tobacco. The frequency of purchase of chewing tobacco is reported high (67.2%) in the older age group than the younger age group (50%). The respondents making more frequent chewing tobacco purchase were more likely to report feelings of strong cravings than those who make less purchase of chewing tobacco. 36.4% reported strong cravings for chewing tobacco and 27.3% reported they never go more than 2 hours without chewing tobacco. 31.8% reported sometimes having been drowsy and 10.2% reported often having been drowsy without chewing tobacco.

Total dependence score is generated by the sum of the questions 2-8 of the Severson Smokeless Tobacco Dependence Scale shows the mean 16.23, median 18, mode 19 range of 4-19 and standard deviation of 3.39 of the Total Dependence Score.

## 4. Discussion

The key findings of the study are the use of tobacco leaf mostly compared to other forms of chewing tobacco. Chewing tobacco is used by the older age group. Tobacco is purchased in over half of the respondents every three days. The results of the study indicates a high use of processed or leaf form of chewing tobacco, over half of the respondents reported strong cravings for the chewing tobacco and a high percentage of respondents reported the use of chewing tobacco within one hour after waking up.

## 5. Conclusion

The study could be developed in the selection of the sample, which would include both males and females to study the dependency on smokeless tobacco. Tobacco cessation projects for black and minority ethnic groups have often received short -term funding, undermining their sustainability and possible strategic contribution (Crosier & McNeill, 2003). UK resident Pakistani, Bangladeshi men should have the opportunity of benefiting from the further development of tobacco control interventions and services that contribute to the improved provision of information about the range of tobacco available and their use. The imams should be given formal training to fulfill potentially vital role in improving access to services for minority Muslim communities (Ali et al., 2005).

**Figure 1:**
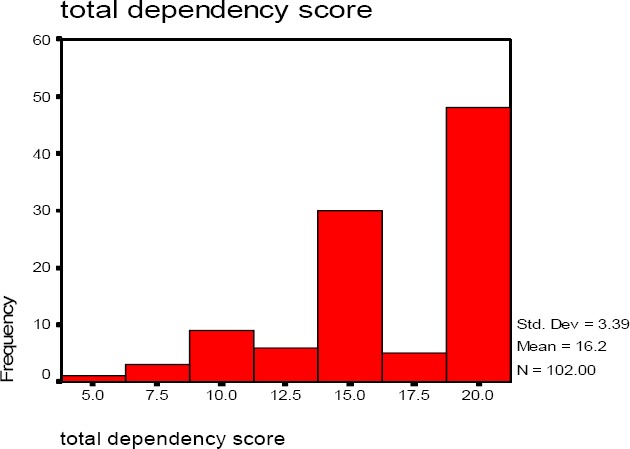
Distribution of the total dependence score in sample of 88 south Asian UK men resident men%

Figure 1 reports the association of frequency distribution and Total Dependence Score in a sample of 88 UK resident South Asian men. The Total Dependence Score of 19 has the highest frequency of 48 (47.1%). Distribution is skewed towards higher score, suggesting high levels of dependency in the respondents.
